# Major threats to early safety after transcatheter aortic valve implantation in a contemporary cohort of real-world patients

**DOI:** 10.1007/s12471-021-01638-8

**Published:** 2021-11-01

**Authors:** D. J. van Ginkel, J. Brouwer, N. D. van Hemert, A. O. Kraaijeveld, B. J. W. M. Rensing, M. J. Swaans, L. Timmers, M. Voskuil, P. R. Stella, J. M. ten Berg

**Affiliations:** 1 Department of Cardiology, St. Antonius Hospital, Nieuwegein, The Netherlands; 2 Department of Cardiology, University Medical Centre Utrecht, Utrecht, The Netherlands

**Keywords:** Transcatheter aortic valve replacement, Complications, Stroke, Haemorrhage, Mortality, Quality improvement

## Abstract

**Introduction:**

Despite considerable advances in the last decade, major adverse events remain a concern after transcatheter aortic valve implantation (TAVI). The aim of this study was to provide a detailed overview of their underlying causes and contributing factors in order to identify key domains for quality improvement.

**Methods:**

This observational, prospective registry included all patients undergoing TAVI between 31 December 2015 and 1 January 2020 at the St. Antonius Hospital in Nieuwegein and the University Medical Centre in Utrecht. Outcomes of interest were all-cause mortality, stroke, major bleeding, life-threatening or disabling bleeding, major vascular complications, myocardial infarction, severe acute kidney injury and conduction disturbances requiring permanent pacemaker implantation within 30 days after TAVI, according to the Valve Academic Research Consortium‑2 criteria.

**Results:**

Of the 1250 patients who underwent TAVI in the evaluated period, 146 (11.7%) developed a major complication. In 54 (4.3%) patients a thromboembolic event occurred, leading to stroke in 36 (2.9%), myocardial infarction in 13 (1.0%) and lower limb ischaemia in 11 (0.9%). Major bleeding occurred in 65 (5.2%) patients, most frequently consisting of acute cardiac tamponade (*n* = 25; 2.0%) and major access-site bleeding (*n* = 21; 1.7%). Most complications occurred within 1 day of the procedure. Within 30 days a total of 54 (4.3%) patients died, the cause being directly TAVI-related in 30 (2.4%). Of the patients who died from causes that were not directly TAVI-related, 14 (1.1%) had multiple hospital-acquired complications.

**Conclusion:**

A variety of underlying mechanisms and causes form a wide spectrum of major threats affecting early safety in 11.7% of patients undergoing TAVI in a contemporary cohort of real-world patients.

**Supplementary Information:**

The online version of this article (10.1007/s12471-021-01638-8) contains supplementary material, which is available to authorized users.

## What’s new?

- We provide a detailed overview of the underlying causes and impact of major complications following transcatheter aortic valve implantation (TAVI), based on a contemporary cohort of real-world patients.
- A variety of mechanisms and causes formed a wide spectrum of major threats affecting early safety in 11.7% of patients undergoing TAVI.
- Most thromboembolic and bleeding complications occurred within 1 day of TAVI. More evidence is needed concerning the optimal periprocedural antithrombotic strategy.
- A substantial number of patients died after a multifactorial course of predominantly hospitalisation-related complications, which resembled the causal chain disease model of geriatric syndromes and may indicate that better patient selection and postprocedural care could improve patient benefit and lower 30-day mortality.

## Introduction

Despite considerable advances in the last decade, major adverse events remain a concern after transcatheter aortic valve implantation (TAVI), as they significantly limit quality of life in the affected patients [1–3]. This is particularly the case, since improvement of quality of life is considered highly important in this fragile, elderly population, with the highest mean age of all cardiac interventions [4, 5]. Therefore, efforts to further reduce these events are likely to yield important clinical benefits [3, 6, 7]. Accordingly, the Netherlands Heart Registry (NHR) collects and publishes data on all cardiac interventions in the Netherlands. In the annual NHR report of 2019, 30-day mortality following TAVI between 2014 and 2018 differed considerably between TAVI centres, varying from 1.9% to 5.7%. The same applied to stroke and vascular complications [5]. This underscores the need for further quality improvement. However, the numbers reported by the NHR do not provide specific starting points for quality improvement. Underlying causes and contributing factors behind these numbers need to be identified first. Therefore, the aim of the present study was to provide a contemporary, in-depth overview of the underlying causes and impact of major complications following TAVI, based on the detailed institutional registries and additional analyses of affected individual cases, in two high-volume centres in the Netherlands.

## Methods

Baseline characteristics, imaging data, procedural characteristics and clinical outcomes were collected in a prospective registry for all patients undergoing TAVI between 31 December 2015 and 1 January 2020 at the St. Antonius Hospital in Nieuwegein and the University Medical Centre in Utrecht. Prior to the procedure, patients underwent a detailed assessment including echocardiography, computed tomography and coronary angiography. Subsequently, all cases were discussed by an interdisciplinary heart team during which the indication for and feasibility of TAVI was established. The electronic patient files of patients who suffered an outcome of primary interest were studied in detail in addition to the analyses of the registry data. This study was approved by the medical ethics committees of both centres; the need for informed consent was waived.

### Outcomes of interest

Outcomes of primary interest were all-cause mortality, stroke, major bleeding, life-threatening or disabling bleeding, major vascular complications, myocardial infarction and acute kidney injury (AKI, stage 2 and 3) within 30 days after TAVI, according to the Valve Academic Research Consortium (VARC)-2 criteria [8]. These were combined in an early safety composite endpoint. Conduction disturbances requiring permanent pacemaker implantation (PPI) were a secondary outcome. Clinical disability following stroke was measured using the modified Rankin scale (mRS) at baseline, the day of stroke diagnosis, the day of discharge and after 90 days, by two authors independently [9]. A disabling stroke is defined as a stroke resulting in a mRS score ≥ 2 at 90 days and an increase of ≥ 1 mRS category from baseline [8]. Haemorrhagic transformation following stroke was considered part of the initial complication and not analysed as a bleeding complication.

### Statistical analysis

Continuous variables were described using mean ± standard deviation or median and interquartile range, in the case of non-normal distribution. Variables were compared using the independent-samples *t*-test or the Mann-Whitney U test, as appropriate. For categorical variables, absolute numbers and percentages were given and group comparisons were performed with the Pearson chi-square test or Fisher’s exact test. Independent predictors of the early safety composite endpoint were identified by first including the parameters in a univariate regression analysis and subsequently entering the significant predictors in a forward stepwise multivariate logistic regression model. A two-tailed *p-*value of < 0.05 was considered statistically significant. Statistical analyses were performed using IBM SPSS Statistics, version 26 (IBM Corp., Armonk, NY, USA).

## Results

**Fig. 1:**
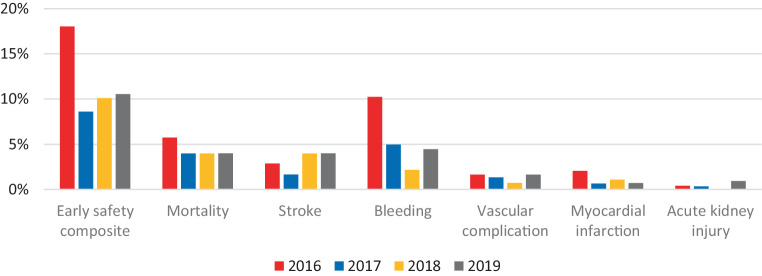
Occurrence of primary endpoints per year

A total of 1250 patients underwent TAVI in the evaluated time period. Baseline characteristics are presented in Table S1 (Electronic Supplementary Material). Patients who developed a major complication (early safety composite endpoint) more frequently had a history of peripheral artery disease (*p* = 0.015) and pulmonary hypertension (*p* = 0.035), compared with patients not suffering a major complication. Additionally, they were in a higher New York Heart Association class (*p* = 0.006), had worse left ventricular ejection fraction (*p* = 0.026) and mitral regurgitation (*p* = 0.020), and a higher Edmonton Frail Scale (EFS) score (*p* = 0.005). Other baseline characteristics were comparable between groups, as shown in Table S1 (Electronic Supplementary Material). In total, 192 major complications occurred in 146 (11.7%) patients. The yearly occurrence of major complications is shown per category in Fig. 1. After multivariate analysis, the EFS remained as the only predictor (odds ratio 1.17 per point EFS increase (95% confidence interval: 1.05–1.30), *p* = 0.003) for occurrence of the early safety composite outcome.

Procedural characteristics are shown in Table S2 (Electronic Supplementary Material). In the majority of patients (88.0%), a transfemoral TAVI was performed. In the remaining patients a transapical (8.1%), direct aortic (2.9%) or transsubclavian (0.5%) approach was used. Major complications more frequently occurred in patients treated with a transapical and direct aortic approach (*p* = 0.001). Median length of postprocedural stay was 6 (4–13) days in those who suffered a major complication as compared to 4 (2–5) days in patients without a major complication (*p* < 0.001). Overall, the median length of postprocedural stay gradually declined from 5 (4–7) days in 2016 to 2 (2–5) days in 2019. Of the patients who suffered a major complication, 60 (42.0%) were discharged home, 43 (30.1%) died, 19 (13.3%) were transferred to the referring hospital, 17 (11.7%) to a (geriatric) rehabilitation clinic and 4 (2.8%) to a nursing home.

### Thromboembolic complications

**Fig. 2:**
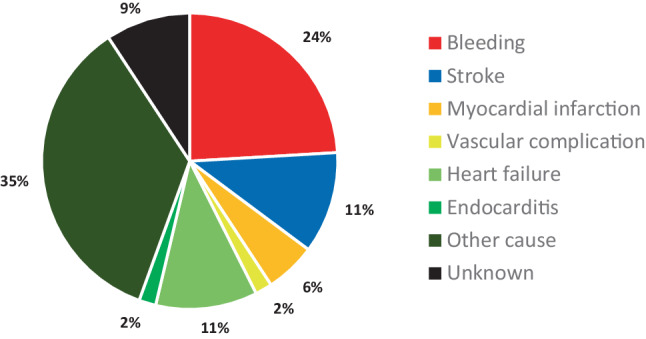
Underlying causes of 30-day mortality

A total of 60 thromboembolic events occurred in 54 (4.3%) patients, leading to stroke in 36 (2.9%), myocardial infarction in 13 (1.0%) and lower limb ischaemia in 11 (0.9%) patients. Most events (83.3%) occurred within 1 day of TAVI. Ten patients (18.5%) who developed a thromboembolic event died within 30 days after TAVI, as depicted in detail in Fig. 2.

#### Stroke

All strokes were ischaemic, with haemorrhagic transformation occurring in three patients. In 24 patients (66.7%) this concerned the anterior cerebral circulation, in nine (25%) the posterior circulation and in three patients (8.3%) multiple vascular territories were affected. This led to dysarthria and aphasia (*n* = 10; 27.7%), hemiparesis (*n* = 8; 22.2%), hemiplegia (*n* = 6; 16.6%) and (hemi)anopsia, balance and coordination disorders (*n* = 4; 11.1%). The median mRS was 1.5 (1–2) before TAVI, 5 (4–5) on the day of the stroke, 4 (3–5) at discharge and 3 (3–5) after 90 days. In 26 (72.2%) patients this led to a mRS of ≥ 2 and an increase of ≥ 1 mRS category at 90 days after stroke onset. These strokes were therefore classified as disabling. The mortality rate at 90 days was 22.2%.

Half of the stroke patients had a history of atrial fibrillation (AF), and in 11 (30.6%) stroke cases AF was documented during hospitalisation. One patient who was diagnosed with new-onset AF 1 day after TAVI suffered a disabling stroke 4 days post-TAVI. He was not treated with oral anticoagulation despite a CHAD_2_S‑VASc score of 4, as AF was considered to be periprocedural. In the 113 (9.0%) patients in whom a cerebral embolic protection device was used, a clinically overt stroke occurred in < 1%. Stroke treatment was conservative in the majority (77.8%) of patients with antithrombotic therapy, which had been initiated before stroke occurrence for other indications in most cases. In four patients endovascular thrombectomy was performed, resulting in partial symptom resolution in two cases. However, in one patient endovascular thrombectomy led to fatal haemorrhagic transformation. Furthermore, five patients were treated with intravenous thrombolysis, resulting in partial symptom resolution in two cases, but treatment was complicated by major access-site bleeding in three of them.

#### Myocardial infarction

Seven of the 13 myocardial infarctions were caused by intraprocedural mechanical coronary artery obstruction, all but one in the left coronary artery. This required emergency percutaneous coronary intervention with left main stenting in five, and emergency coronary artery bypass grafting and surgical aortic valve replacement in two patients. A postprocedural myocardial infarction occurred in six cases. One case occurred due to anticoagulation reversal because of acute cardiac tamponade. In the remaining five cases the event occurred ≥ 72 h after TAVI and these were therefore classified as spontaneous myocardial infarctions.

#### Lower limb ischaemia

Ipsilateral lower limb ischaemia was caused by iliac or femoral occlusion in five, and occlusive iliac or femoral dissection in six patients. Eventually, all 11 patients underwent surgical thrombectomy or reconstruction, as two patients who received endovascular treatment remained symptomatic and a patient initially treated conservatively developed invalidating claudication symptoms. This was fatal in one case, a patient who developed ventricular fibrillation during surgery and subsequently died from cardiogenic shock in the intensive care unit.

### Bleeding and vascular complications

A total of 66 major bleeding, life-threatening or disabling bleeding events occurred in 65 (5.2%) patients, of which acute cardiac tamponade (38.5%) and major access-site bleeding (32.3%) were the most frequent. Details about the occurrence at other bleeding sites can be found in Tab. 1. Thirteen patients (20.0%) who developed a bleeding event died within 30 days after TAVI, as depicted in Fig. 2.

**Table 1:** Overview of primary outcomes. For the main categories the number of cases is shown, with the incidence as a percentage in parentheses

Primary outcomes (30 days)	*N* = 192
*Stroke*	36 (2.9)
– Disabling	26
– Non-disabling	10
*Myocardial infarction*	13 (1.0)
– Periprocedural	10
– Spontaneous	3
*Major non-bleeding, vascular complications*	17 (1.3)
– Acute limb ischaemia	11
– Aortic dissection	4
– Ventricular perforation^a^	2
*Major, life-threatening or disabling bleeding*	66 (5.2)
– Cardiac tamponade	25
– Haemothorax	3
– Gastrointestinal	4
– Intra-abdominal bleeding	3
– Retroperitoneal bleeding	4
– Access site	21
– Other	6
*All-cause mortality*	54 (4.3)
– TAVI related	30
– Other cause	19
– Unknown	5
*Severe acute kidney injury*	6 (0.5)
– Stage 2	3
– Stage 3	3
Early safety composite outcome	146 (11.7)

*TAVI* transcatheter aortic valve implantation

^a^In two cases ventricular perforation occurred that did not lead to serious bleeding and cardiac tamponade. Consequently these cases were classified as major non-bleeding, vascular complications

#### Acute cardiac tamponade

Pericardial bleeding was caused by left ventricular guidewire perforation (*n* = 8; 32.0%), (sub)annular rupture (*n* = 7; 28.0%), right ventricular perforation after pacemaker lead insertion (*n* = 4; 16.0%) or left ventricular free-wall rupture (*n* = 1; 4.0%). In five patients (20.0%), the underlying cause remained unascertained. Pericardiocentesis was performed in 18 (72.0%) and surgical drainage and correction in seven patients (28.0%). No emergency surgery was performed based on a pre-agreed treatment limitation or lack of surgical options in the remaining cases. The in-hospital mortality rate of this subset of patients developing a tamponade was 44.0%.

#### Major access-site bleeding

Major access-site bleeding occurred most commonly in the groin (*n* = 18; 85.7%) after transfemoral TAVI. Furthermore, two (9.5%) transapically treated patients developed a haemothorax and one developed aortic bleeding following direct aortic access. These access-site bleedings were classified as major or life-threatening based on ≥ 3.0 g/dl decrease in haemoglobin level (*n* = 3), requiring transfusion of ≥ 2 units (*n* = 7) or requiring surgery (*n* = 11).

#### Vascular complications

In four patients, aortic dissection occurred (0.3%); two type A and two type B dissections, all without coronary, aortic arch or abdominal vessel involvement. They were treated conservatively with strict blood pressure regulation and survived the 30-day follow-up. In two transapically treated patients, ventricular perforation occurred after pacemaker lead insertion. This did not lead to a serious bleeding event, as it could be sutured rapidly.

### Other complications

#### Acute kidney injury

Six patients developed severe AKI after TAVI, three of whom had stage 2 and three stage 3 according to the Acute Kidney Injury Network system. In all of these patients, baseline estimated glomerular filtration rate was below 60 ml/min per 1.73 m^2^. AKI never occurred alone and was part of a multifactorial course in all patients, including stroke (*n* = 3), bleeding (*n* = 4) and PPI due to total atrioventricular (AV) block (*n* = 1). Half of these patients did not survive the 30-day follow-up period.

#### Conduction disturbances requiring PPI

A total of 138 (11.0%) patients developed a conduction disturbance leading to PPI. In 76 (55.1%) patients the indication for PPI was established within 2 days after TAVI. The mean time period between TAVI and PPI was 5 days. Indications for PPI were third-degree AV block in 98 (71.0%), high-degree AV block in 20 (14.5%), progressive first- degree AV block with bundle branch block in 11 (8.0%), alternating bundle branch block and sick sinus syndrome in 4 (2.9%) patients each, and reflex asystolic syncope in one patient. The majority (*n* = 136, 98.6%) survived the 30-day follow-up period.

### Mortality

A total of 54 (4.3%) patients deceased within 30 days after TAVI. Ten patients (18.5%) died during the procedure, 26 (48.1%) post-TAVI during hospitalisation and 18 (33.3%) after discharge. Deaths were especially prevalent (*n* = 11; 10.9%) in patients treated by transapical approach. Most deaths (55.6%) were caused by TAVI-related complications, as described in the previous sections and shown in Fig. 2. Furthermore, one patient died from *Staphylococcus aureus* endocarditis of the recently implanted aortic valve prosthesis and six patients due to progressive heart failure. One of them died during the procedure due to concomitant severe mitral regurgitation, and another due to aortic valve regurgitation, after predilatation and valve pop-out. The remaining deaths from progressive heart failure were based on severe paravalvular leakage (*n* = 2), severe mitral regurgitation (*n* = 1) and lack of contractile reserve in all patients. A paravalvular leakage closure attempt was made in one patient; the others were deemed too weak for further intervention.

A substantial number of patients (*n* = 19; 35.2%) died from other not directly TAVI-related causes. A case of fatal arrhythmia was caused by hyperkalaemia. Four patients died from non-coronary vascular conditions, i.e. intestinal ischaemia in two patients, a massive pulmonary embolism and a spontaneous subdural haematoma. A combination of factors—such as hospital-acquired pneumonia, urinary tract infection, sepsis, delirium, AF, fluid retention and renal insufficiency—contributed to the eventual fatal outcome in 14 cases. The underlying cause remained unknown in five (9.3%) patients, who died after discharge.

## Discussion

This study shows that a spectrum of individually rare complications together still result in a relatively high adverse event rate in patients currently undergoing TAVI. The incidences of the evaluated events were fairly comparable with recent reports of other Dutch [10], European [11, 12], American [12, 13] and Asian [14] TAVI registries of real-world patients. Rates of stroke and mortality were, however, on the high end of the spectrum, while bleeding complications were on the low end [10–14].

From a previously described bimodal pattern of stroke occurrence in the 30 days following TAVI, i.e. within the first 24 h and in a late phase of > 10 days after TAVI, only the first peak was observed in this cohort [7, 15]. Despite considerable variability in clinical presentation and symptom severity, the major negative prognostic impact of stroke following TAVI as shown by the high disability and mortality rates is consistent with the body of literature on this topic [15]. A remarkable number of stroke patients also suffered from AF. A large meta-analysis on this topic demonstrated an incidence of new-onset AF after TAVI of 17.5%. Interestingly, pre-existing AF did not, but new-onset AF did increase the risk of stroke significantly shortly after TAVI [16]. This seems to underline the importance of adequate oral anticoagulation in these patients. In contrast to a recent overview of anecdotal evidence regarding endovascular stroke treatment following TAVI, in which no complications post-recanalisation were observed, our data illustrate the high and potentially fatal bleeding risk associated with thrombolytic and endovascular stroke treatment post-TAVI [17]. The efficacy of cerebral embolic protection device usage seems promising based on our observational data. However, the current evidence on this topic is inconclusive and suffers from both validity and precision limitations [18]. Therefore, the results of the large Stroke PROTECTion With SEntinel During Transcatheter Aortic Valve Replacement trial (NCT04149535) are awaited expectantly and will provide a well-substantiated view on the role of cerebral embolic protection devices in TAVI.

Although stroke is often considered the most feared TAVI complication, major bleeding, life-threatening and disabling bleeding complications formed the largest complication and mortality category in this cohort. Especially acute cardiac tamponade negatively impacted survival after TAVI. Technical improvements may further lower the incidence of these devastating complications [19, 20]. Wang et al. found a strong association between major and life-threatening bleeding and 30-day mortality in a meta-analysis on this topic and identified pre-existing AF and transapical access as the most important risk factors of post-TAVI bleeding [21]. In line with previous studies, the authors suggest as the underlying mechanism that the presence of AF should be considered a surrogate for oral anticoagulation use, which is commonly administered in high-risk patients with AF undergoing TAVI [6, 21]. The recently published results of cohort B of the POPular TAVI trial provided more insight into the optimal postprocedural antithrombotic strategy in this subgroup of TAVI patients [22]. However, despite the fact that most bleeding and thromboembolic events occur within 1 day of TAVI, less is known about the optimal periprocedural anticoagulation strategy. Interestingly, interruption of oral anticoagulation has recently been associated with an increased risk of thromboembolic events during TAVI [23]. The results of the Periprocedural Continuation Versus Interruption of Oral Anticoagulant Drugs During Transcatheter Aortic Valve Implantation (POPular PAUSE TAVI) trial (NCT04437303) will provide more evidence on this topic.

Beside thromboembolic and bleeding complications, a substantial number of patients died from causes that were not directly TAVI-related (Fig. 1). After an in-depth evaluation, the common thread in these cases was a multifactorial course of hospitalisation-related complications such as hospital-acquired pneumonia and delirium, which eventually led to the fatal outcome. This resembled the causal chain disease model of geriatric syndromes [24] and may indicate that better patient selection could improve patient benefit and lower 30-day mortality. This is emphasised by the finding of a higher EFS score as an independent predictor of the early safety composite endpoint. To improve this selection process, adequate patient-centred risk scores, indicating when TAVI should no longer be performed as it would be futile, need to be studied further [25]. This is particularly the case, since existing prediction models of 30-day mortality after TAVI have recently been proven to have a poor predictive performance in the Dutch population [10]. This is equivalently reflected by the moderate c‑statistic (0.64) of the NHR model, predicting the expected 30-day mortality per TAVI centre adjusted for specific patient characteristics. This moderate c‑statistic is assumed to be caused by the lack of essential predictors, less power as compared with the NHR percutaneous coronary intervention model and more heterogeneity in the entire care process between TAVI centres [8]. Regarding this last factor, improvement in postprocedural care was identified as another domain for quality improvement, as the majority of the underlying complications in the multifactorial course described above are known to be hospitalisation related. More detailed registries regarding TAVI care processes are needed to identify how this can be optimised in clinical practice.

### Strengths and limitations

In addition to the numbers of TAVI complications uniquely published by NHR, the present study unravelled their underlying causes, factors and impact. Beside this illuminating picture itself, the main lesson of this analysis is that although individually these complications are rare, together they form a heavy burden affecting a substantial proportion of TAVI patients.

Inter-registry differences were a limitation of this study, hampering a more detailed evaluation of other (post)procedural and baseline characteristics. In addition, efficacy and long-term outcomes were not registered in a similar manner, which could have provided additional viewpoints for quality improvement. Furthermore, missing data was a concern for some variables, since not all patients were referred for geriatric consultation and clinical use of risk scores changed during the studied period. Nevertheless, the evaluated outcomes were based on identical criteria in both registries and were available for all patients.

## Conclusion

A variety of underlying mechanisms and causes formed a wide spectrum of major threats affecting early safety in 11.7% of patients undergoing TAVI and led to serious morbidity and mortality in a contemporary cohort of real-world TAVI patients. To enhance patient benefit, periprocedural antithrombotic therapy, patient selection and postprocedural care were identified as key domains for quality improvement.

## Supplementary Information

**Table S1. **Baseline table. *NYHA* New York Heart Association, *COPD* chronic obstructive pulmonary disease, *CABG* coronary artery bypass grafting, *SAVR* surgical aortic valve replacement, *PPI* permanent pacemaker implantation, *LVEF* left ventricular ejection fraction.

**Table S2. **Procedural details.

## References

[1] Vranckx P, Windecker S, Welsh RC, Valgimigli M, Mehran R, Dangas G (2017). Thrombo-embolic prevention after transcatheter aortic valve implantation. Eur Heart J.

[2] Winter MP, Bartko P, Hofer F (2020). Evolution of outcome and complications in TAVR: a meta-analysis of observational and randomized studies. Sci Rep.

[3] Arnold SV, Zhang Y, Baron SJ (2019). Impact of short-term complications on mortality and quality of life after transcatheter aortic valve replacement. JACC Cardiovasc Interv..

[4] Vendrik J, Baan J (2020). Meta-analysis of randomised trials compares mortality after transcatheter versus surgical aortic valve replacement. Neth Heart J.

[5] Stichting Nederlandse Hart Registratie. Rapportage 2019.. https://nederlandsehartregistratie.nl/wp-content/uploads/2020/01/NHR-Rapportage-2019-per-spread-230120.pdf. Accessed 7 Oct 2020.

[6] Généreux P, Cohen DJ, Mack M (2014). Incidence, predictors, and prognostic impact of late bleeding complications after transcatheter aortic valve replacement. J Am Coll Cardiol..

[7] Davlouros PA, Mplani VC, Koniari I, Tsigkas G, Hahalis G (2018). Transcatheter aortic valve replacement and stroke: a comprehensive review. J Geriatr Cardiol.

[8] Kappetein AP, Head SJ, Généreux P (2012). Updated standardized endpoint definitions for transcatheter aortic valve implantation: the Valve Academic Research Consortium‑2 consensus document (VARC-2). Eur J Cardiothorac Surg.

[9] UK-TIA Study Group. United Kingdom transient ischaemic attack (UK-TIA) aspirin trial (1988). UK-TIA Study Group. United Kingdom transient ischaemic attack (UK-TIA) aspirin trial: interim results. UK-TIA Study Group. Br Med J (Clin Res Ed).

[10] Al-Farra H, Abu-Hanna A, de Mol BAJM (2020). External validation of existing prediction models of 30-day mortality after transcatheter aortic valve implantation (TAVI) in the Netherlands Heart Registration. Int J Cardiol.

[11] Pepe M, Corcione N, Biondi-Zoccai G (2020). Comparison of outcomes of transcatheter aortic valve implantation in patients ≥85 years versus those <85 years. Am J Cardiol.

[12] Matsuda Y, Nai Fovino L, Giacoppo D (2021). Association between surgical risk and 30-day stroke after transcatheter versus surgical aortic valve replacement: a systematic review and meta-analysis. Catheter Cardiovasc Interv.

[13] Masha L, Vemulapalli S, Manandhar P (2020). Demographics, procedural characteristics, and clinical outcomes when cardiogenic shock precedes TAVR in the United States. JACC Cardiovasc Interv..

[14] Takagi K, Naganuma T, Tada N (2020). The Predictors of peri-procedural and sub-acute cerebrovascular events following TAVR from OCEAN-TAVI Registry. Cardiovasc Revasc Med.

[15] Armijo G, Nombela-Franco L, Tirado-Conte G (2018). Cerebrovascular events after transcatheter aortic valve implantation. Front Cardiovasc Med.

[16] Sannino A, Gargiulo G, Schiattarella GG (2016). A meta-analysis of the impact of pre-existing and new-onset atrial fibrillation on clinical outcomes in patients undergoing transcatheter aortic valve implantation. EuroIntervention.

[17] Suleiman S, Szirt R, Coughlan JJ (2020). Mechanical thrombectomy for transcatheter aortic valve insertion (TAVI)-related periprocedural stroke: current literature and future directions. EMJ Int Cardiol.

[18] Lansky A, Pietras C, Shah T (2020). Cerebral embolic protection: burden of proof. JACC Cardiovasc Interv..

[19] Scarsini R, De Maria GL, Joseph J (2019). Impact of complications during transfemoral transcatheter aortic valve replacement: how can they be avoided and managed?. J Am Heart Assoc.

[20] Tanaka M, Yanagisawa R, Yashima F (2020). A novel technique to avoid perforation of the right ventricle by the temporary pacing lead during transcatheter aortic valve implantation. Cardiovasc Interv Ther.

[21] Wang J, Yu W, Jin Q (2017). Risk factors for post-tavi bleeding according to the varc‑2 bleeding definition and effect of the bleeding on short-term mortality: a meta-analysis. Can J Cardiol.

[22] Nijenhuis VJ, Brouwer J, Delewi R (2020). Anticoagulation with or without clopidogrel after transcatheter aortic-valve implantation. N Engl J Med..

[23] Brinkert M, Keller LS, Moriyama N (2019). Safety and efficacy of transcatheter aortic valve replacement with continuation of oral anticoagulation. J Am Coll Cardiol..

[24] Inouye SK, Studenski S, Tinetti ME, Kuchel GA (2007). Geriatric syndromes: clinical, research, and policy implications of a core geriatric concept. J Am Geriatr Soc.

[25] Allen C, Patterson T, Redwood S, Prendergast B (2020). Frailty in patients undergoing transcatheter aortic valve replacement: frequently measured, seldom managed. JACC Cardiovasc Interv..

