# Supplementary material for: Major threats to early safety after transcatheter aortic valve implantation in a contemporary cohort of real-world patients
Source: Neth Heart J. 2021 Nov 1;29(12):632–42. doi: 10.1007/s12471-021-01638-8 (PMC8630308; doi:10.1007/s12471-021-01638-8)
Supplement: Supplementary file 1 — Table S1. Baseline table. NYHA New York Heart Association, COPD chronic obstructive pulmonary disease, CABG coronary artery bypass grafting, SAVR surgical aortic valve replacement, PPI permanent pacemaker implantation, LVEF left ventricular ejection fraction. [file 12471_2021_1638_MOESM1_ESM.docx]

**Table S1.** Baseline table. *NYHA* New York Heart Association, *COPD* chronic obstructive pulmonary disease, *CABG* coronary artery bypass grafting, *SAVR* surgical aortic valve replacement, *PPI* permanent pacemaker implantation, *LVEF* left ventricular ejection fraction.

| **Baseline characteristics** | **Major complication**  **(*n*=146)** | **No major complication**  **(*n*=1104)** | ***P*** |
| --- | --- | --- | --- |
| Age ± SD | 80.8 ± 6.5 | 80.4 ± 6.8 | 0.45 |
| Male – *n* (%) | 69 (47.3) | 560 (50.7) | 0.43 |
| Body mass index ± SD | 26.0 ± 4.6 | 26.8 ± 5.0 | 0.05 |
| NYHA class – *n* (%)  I  II  III  VI | 8 (5.5)  38 (26.2)  85 (58.6)  14 (9.7) | 83 (7.6)  410 (37.4)  549 (50.1)  53 (4.8) | <0.01 |
| Hypertension – *n* (%) | 102 (69.9) | 787 (71.4) | 0.70 |
| Diabetes mellitus – *n* (%) | 33 (22.6) | 288 (26.1) | 0.37 |
| Coronary artery disease – *n* (%) | 82 (57.3) | 550 (49.7) | 0.09 |
| Atrial fibrillation – *n* (%) | 55 (37.7) | 398 (36.1) | 0.71 |
| Peripheral artery disease – *n* (%) | 36 (24.7) | 182 (16.5) | 0.02 |
| Previous stroke – *n* (%) | 22 (15.1) | 111 (10.1) | 0.07 |
| Pulmonary hypertension – *n* (%) | 17 (12.7) | 76 (7.4) | 0.04 |
| COPD – *n* (%) | 26 (17.8) | 193 (17.5) | 0.93 |
| Previous CABG – *n* (%) | 18 (12.3) | 184 (16.7) | 0.18 |
| Previous SAVR – *n* (%) | 8 (5.6) | 72 (6.5) | 0.67 |
| Previous PPI– *n* (%) | 14 (9.6) | 101 (9.1) | 0.86 |
| LVEF – *n* (%)  >50%  31-50%  ≤30% | 77 (53.1)  49 (33.8)  19 (13.1) | 668 (60.7)  354 (32.2)  78 (7.1) | 0.03 |
| Mitral regurgitation – *n* (%)  None/trace  Mild  Moderate  Severe | 36 (25.7)  63 (45.0)  28 (20.0)  13 (9.3) | 317 (30.1)  496 (47.1)  200 (19.0)  39 (3.7) | 0.02 |
| Aortic valve area ± SD | 0.74 ± 0.22 | 0.78 ± 0.22 | 0.10 |
| Mean pressure gradient ± SD | 42.0 ± 17.5 | 39.6 ± 20.6 | 0.15 |
| Max pressure gradient ± SD | 66.9 ± 25.4 | 64.7 ± 24.1 | 0.35 |
| EuroSCORE II (IQR) | 5.2 (5.1) | 4.5 ± (3.8) | 0.13 |
| Edmonton Frail Scale (IQR) | 4.7 (2.8) | 3.7 (2.3) | <0.01 |
