# Supplementary material for: Major threats to early safety after transcatheter aortic valve implantation in a contemporary cohort of real-world patients
Source: Neth Heart J. 2021 Nov 1;29(12):632–42. doi: 10.1007/s12471-021-01638-8 (PMC8630308; doi:10.1007/s12471-021-01638-8)
Supplement: Supplementary file 2 — Table S2. Procedural details. [file 12471_2021_1638_MOESM2_ESM.docx]

**Table S2** Procedural details

| **Procedural details** | **Major complication**  **(*n*=146)** | **No major complication**  **(*n*=1104)** | ***P*** |
| --- | --- | --- | --- |
| Access approach – *n* (%)  Transfemoral  Transapical  Direct aortic  Trans-subclavian | 116 (79.5)  21 (14.4) 9 (6.2)  0 (0.0) | 984 (89.7)  80 (7.3)  27 (2.5)  6 (0.5) | <0.01 |
| Prosthesis type – *n* (%)  Edwards Sapien XT, 3, 3 Ultra  Metronic CoreValve Evolut, R, Pro  Symetis Acurate  Lotus Valve System  JenaValve  Other | 92  64 (43.8)  50 (34.2)  12 (8.2)  12 (8.2)  6 (4.1)  1 (0.7) | 480 (43.5)  389 (35.2)  149 (13.5)  47 (4.3)  14 (1.3)  21 (1.9) | 0.01 |
| Local anaesthesia – *n* (%) | 79 (54.5) | 680 (62.8) | 0.07 |
| Valve size (mm) ± SD | 27.1 ± 3.0 | 27.1 ± 2.9 | 0.85 |
| Predilation – *n* (%) | 62 (43.1) | 493 (44.9) | 0.68 |
| Postdilation – *n* (%) | 34 (23.8) | 257 (23.5) | 0.94 |
| Median length of postprocedural stay (IQR) | 6.0 (4-13) | 4.0 (2-5) | <0.01 |
